# The Seybert Commission

**Published:** 1887-11

**Authors:** 


### THE SEYBERT COMMISSION.

The late report of the above named committee of reverend and miscellaneous *quasi* experts, explaining how they contrived to avoid the duty assigned them to make a fair investigation of modern spiritualism, its philosophy and phenomena, has been very thoroughly exposed and shown up by Prof, Henry Kiddle, of New York City, as a committee of one of the *American Spiritualist Alliance,* the full text whereof is contained in the Oct. 15th and 22d numbers of the well known and widely circulated, Banner of Light. We have only space for the concluding paragraphs contained in Prof. Kiddles summary of his very able and exhaustive report, and by him numbered from one to six, they are as follows:

1. There has been no sufficient or proper compliance, on the part of the Trustees of the University of Pennsylvania, with the terms imposed by the founder of the Adams Seybert Chair and the conditions prescribed by him, and agreed to by the University on the acceptance of the fund of $60,000 bequeathed by Mr. Seybert, on the condition that the "incumbent of the Chair," aided or not as he desired, by a "Committee of the Faculty," should make the investigation.

2. It was a gross violation of those conditions, after the original appointment of five members of the faculty to act as a commission, to appoint five additional persons who were not members of the Faculty; and especially to appoint four of these persons, not connected with the University at all, several months after the investigation had commenced.

3. The Commission were obligated to investigate the claims of modern spiritualism, not merely as to its phenomenal basis but as a system of "morals, religion, and philosophy;" and this they have not even commenced to do; but have only, in a most unfair, superficial, inconsiderate, and we might almost say irrational, manner, examined the manifestations of a few mediums, whom they have, most unjustly, according to their own record, held up to public scorn and indignation. Under such circumstances, to rush into print with conclusions so lame, imperfect and ill-founded, should subject them to the censure of every impartial and intelligent mind, as we doubt not it eventually will.

4. They have presented to the public a series of statements, called a Report, crude, imperfect, sweepingly condemnatory, and wholly unscientific, neither correctly representing the facts of their own investigation, as a commission, nor making those distinctions and discriminations as to incidents, principles, and methods which a proper knowledge of the subject would have dictated.

5. Their report is contradicted in many essential particulars by the minutes of their proceedings, which they have chosen to keep in the background, and away from the general reader, by inserting them in an appendix.

6. Instead of conducting the investigation by sub-committees, whose carefully constructed reports could have been attested by the signatures of those making them, they have been guilty of the gross impropriety of presenting a report signed by ten persons, which contains statements that only three or four could truthfully attest. In this way they have misled the public, and especially the newspaper press, and given a seeming importance to their investigation and the report which they do not really possess.

Our readers will call to mind the decision of the Buffalo committee of physicians, which held an inquest upon the *raps* produced through the instrumentality of the "Fox girls" many years ago. This committee reported that it was all a trick and gave an explanation of the *modus operandi* so wanting in common sense, that, to use a homely figure, "The dog it was that died." But the raps have made their way round the world, and appealing to the intelligent and unprejudiced, have opened the way to a higher conception of life here and its inevitable continuance in other states of existence.

There is no class quite so conservative as clergymen, and next in order of unprogressive dogmatists, come old school physicians. In proof of it, we have only to trace their slow and reluctant steps as they have been fairly forced into the light of newly acquired knowledge.

Had those great inventions and discoveries which have shed new light upon the world, been left to committees composed exclusively of these pretentious gentlemen, there would have been but small progress towards the sublime heights from which the tyro of our common schools may now look down upon the bewildering darkness of an age when the exercise of unrecognized accomplishments, were accounted a special gift of the evil one, to be finally compensated by an eternity of suffering. Fortunately it is not by your leave, gentlemen of the Seybert Commission, that the *world moves*.

